# ALTRUISTIC AGING: ON THE MORALITY OF “AGING WELL”

**DOI:** 10.1093/geroni/igac059.1150

**Published:** 2022-12-20

**Authors:** Anne Barrett, Katia Vecchione

**Affiliations:** Florida State University, Tallahassee, Florida, United States; University of Trento, Trento, Trentino-Alto Adige, Italy

## Abstract

A dominant aging narrative emphasizes what individuals should do to age well, namely remain active and productive. Underlying these promotional messages, however, are others about what should be avoided. At their core is a proscription against becoming dependent, thus “burdensome.” To critically examine this aging narrative, we develop the concept of “altruistic aging,” which captures a cultural expectation that older adults adopt a selfless concern for the well-being of others. We use data from interviews with 28 Italians aged 65 and older to illustrate how the goal of altruistic aging shapes older adults’ behaviors in the present and their framing of care options in the future. It motivates physical and social activity, as well as healthy eating – all aimed, in part, at extending the duration of one’s self-sufficient years. Altruistic aging also involves expunging the idea of ever living with one’s children and emphasizing the benefits of nursing homes. This observation suggests that altruistic aging may be constructed as a familial duty, particularly in nations like Italy with family-centered models of care. Our analyses reveal that taken-for-granted, and culturally celebrated, orientations toward aging may mask an internalization of ageism that devalues the self to the point of selflessness.

